# Cardiac PI3K p110α attenuation delays aging and extends lifespan

**DOI:** 10.15698/cst2022.08.270

**Published:** 2022-08-08

**Authors:** Mahmoud Abdellatif, Tobias Eisenberg, Alexander Martin Heberle, Kathrin Thedieck, Guido Kroemer, Simon Sedej

**Affiliations:** 1 Department of Cardiology, Medical University of Graz, 8036 Graz, Austria.; 2 Centre de Recherche des Cordeliers, Equipe labellisée par la Ligue contre le cancer, Université de Paris, Sorbonne Université, Inserm U1138, Institut Universitaire de France, Paris 75006, France.; 3 Metabolomics and Cell Biology Platforms, Institut Gustave Roussy, Villejuif 94805, France.; 4 BioTechMed Graz, 8010 Graz, Austria.; 5 Institute of Molecular Biosciences, NAWI Graz, University of Graz, 8010 Graz, Austria.; 6 Field of Excellence BioHealth, University of Graz, 8010 Graz, Austria.; 7 Institute of Biochemistry and Center for Molecular Biosciences Innsbruck, University of Innsbruck, Innsbruck 6020, Austria.; 8 Department of Pediatrics, Section Systems Medicine of Metabolism and Signalingg, University of Groningen, University Medical Center Groningen, Groningen 9700 RB, The Netherlands.; 9 Department for Neuroscience, School of Medicine and Health Sciences, Carl von Ossietzky University Oldenburg, Oldenburg 26129, Germany.; 10 Institut du Cancer Paris CARPEM, Department of Biology, Hôpital Européen Georges Pompidou, AP-HP, Paris 7015, France.; 11 Institute of Physiology, Faculty of Medicine, University of Maribor, 2000 Maribor, Slovenia.

**Keywords:** PI3K, IGF1, insulin signaling, cardiomyopathy, heart failure, aging, autophagy, mitochondrial dysfunction

## Abstract

Phosphoinositide 3-kinase (PI3K) is a key component of the insulin signaling pathway that controls cellular me-tabolism and growth. Loss-of-function mutations in PI3K signaling and other downstream effectors of the insulin signaling pathway extend the lifespan of various model organisms. However, the pro-longevity effect appears to be sex-specific and young mice with reduced PI3K signaling have increased risk of cardiac disease. Hence, it remains elusive as to whether PI3K inhibition is a valid strategy to delay aging and extend healthspan in humans. We recently demonstrated that reduced PI3K activity in cardiomyocytes delays cardiac growth, causing subnormal contractility and cardiopulmonary functional capacity, as well as increased risk of mortality at young age. In stark contrast, in aged mice, experi-mental attenuation of PI3K signaling reduced the age-dependent decline in cardiac function and extended maximal lifespan, suggesting a biphasic effect of PI3K on cardiac health and survival. The cardiac anti-aging effects of reduced PI3K activity coincided with enhanced oxida-tive phosphorylation and required increased autophagic flux. In humans, explanted failing hearts showed in-creased PI3K signaling, as indicated by increased phos-phorylation of the serine/threonine-protein kinase AKT. Hence, late-life cardiac-specific targeting of PI3K might have a therapeutic potential in cardiac aging and related diseases.

Life expectancy continues to rise worldwide, and so does the prevalence and socioeconomic burden of age-related chronic diseases. Amongst these, cardiovascular disorders remain the leading cause of lost healthy life years and ensuing mortality. Thus, defining the molecular and cellular mechanisms dictating the pace of cardiac aging is required for the development of novel interventions that might improve outcomes in cardiovascular disease, beyond the avoidance of traditional risk factors. The inhibition of insulin/insulin-like growth factor-1 signaling (IIS) pathway is an evolutionary conserved mechanism to delay organismal aging. Loss-of-function mutations in key components of the IIS pathway extend the lifespan in various organisms, ranging from yeast to rodents [11], [2]. However, the magnitude of such anti-aging effect appears to depend on sex and the genetic background of tested animals [3]. Since IIS is required for normal growth and metabolism, further studies – on druggable targets of the IIS pathway and preferably in a cell type-specific fashion – must determine whether inhibiting IIS pathway is a valid and safe strategy to compress late-life morbidity in humans. In this respect, aged mice with reduced cardiac activity of phosphoinositide 3-kinase (PI3K), a key downstream effector of the IIS pathway, exhibit preserved cardiac function and reduced expression of senescence markers [4]. By contrast, young mice with cardiomyocyte-specific suppression of PI3K are more prone to dilated and ischemic cardiomyopathy, at least when surgically induced at a young age [5], [6]. In order to resolve these disparate findings, we performed a comprehensive long-term study, in which we assessed cardiac health and survival of two transgenic mouse models with increased or reduced cardiac PI3K signaling throughout the course of life [7].

We observed that mice harbouring an inactive PI3K p110α isoform specifically in cardiac myocytes (dnPI3K) show delayed cardiac growth, subnormal contractility, and attenuated cardiopulmonary functional capacity, leading to an abnormally increased risk of mortality during early life. In contrast, aged dnPI3K mice displayed an attenuated age-dependent decline in cardiac systolic and diastolic functions, preserved cardiac functional reserve, reduced cardiac remodelling as well as improved myocardial bioenergetics, which were associated with extended survival during late-life stages. Mechanistically, delayed cardiac aging in dnPI3K mice coincided with activated autophagic flux in the heart. In fact, autophagy inhibition using the lysosomotropic agent hydroxychloroquine abolished most of the benefits observed in aged dnPI3K hearts, indicating that functional autophagy has a causal role in the late-life cardioprotective effects of reduced PI3K activity.

Conversely, increased IIS-PI3K signaling in mice overexpressing the human insulin-like growth factor-1 in cardiac myocytes (IGF1R^*tg*^) led to augmented heart growth, supranormal contractile function and increased exercise capacity. However, aged IGF1R^*tg*^ mice developed signs of accelerated cardiac aging, as indicated by reduced contractility and exacerbated diastolic dysfunction, leading to effort intolerance, compromised cardiopulmonary functional capacity, lung congestion and shorter lifespan. Aged IGF1R^*tg*^ mice also exhibited increased left ventricular fibrosis as well as severe left atrial remodelling, suggesting an age-related shift from physiological hypertrophy towards hypertrophic cardiomyopathy. Premature cardiac aging in IGF1R^*tg*^ mice correlated with mitochondrial dysfunction and reduced anti-oxidative potential, likely due to blocked cardiac autophagy. In support of this notion, reduced autophagic flux occurred before the development of these deleterious cardiac effects, while reactivating autophagy by the caloric restriction mimetic spermidine prevented the deterioration of oxidative phosphorylation and stress resistance mechanisms, thereby preserving cardiac function and protecting aged IGF1Rtg mice from heart failure [8].

Our findings are likely clinically relevant because we observed cardiac IGF1R overexpression and increased phosphorylation of the serine/threonine-protein kinase AKT in left ventricular samples of patients with non-ischemic dilated cardiomyopathy, denoting activated cardiac IIS-PI3K signaling in heart failure. Downstream to PI3K, we detected increased mammalian target of rapamycin (MTOR)-dependent inhibitory phosphorylation of unc-51 like autophagy activating kinase 1 (ULK1) [9]. Intriguingly, and unlike failing human hearts, donors with compensated hypertrophy (i.e., preserved function, despite increased remodelling) showed no signs of increased IIS-PI3K signaling as compared to age-matched controls with no history of cardiac disease, suggesting a potential protective role of IIS-PI3K inhibition.

In sum, age appears to be a key determining factor that defines the role of PI3K signaling in the heart. At young age, increased activity of the IIS-PI3K axis is required for normal cardiac growth and development. However, later in life reduced PI3K activity confers anti-aging effects, likely through prioritizing cardioprotective quality control mechanisms, such as autophagy, over superfluous cardiac growth (**Fig. 1**). Such a biphasic relationship between cardiac health and IIS-PI3K signaling activity reconciles previous opposing studies [6], [6], and suggests that partial inhibition of PI3K might be worth considering for the treatment of heart failure and other age-related chronic diseases – albeit at a much lower and safer dosage than that applied in cancer therapy [10], [11]. Alternatively, emerging autophagy inducers and caloric restriction mimetics, like spermidine and nicotinamide adenine dinucleotide (NAD^+^) precursors [8], [12], [13], [14], might offer a readily-available, and perhaps safer, strategy for immediate testing in cardiac patients [15].

**Figure 1:**
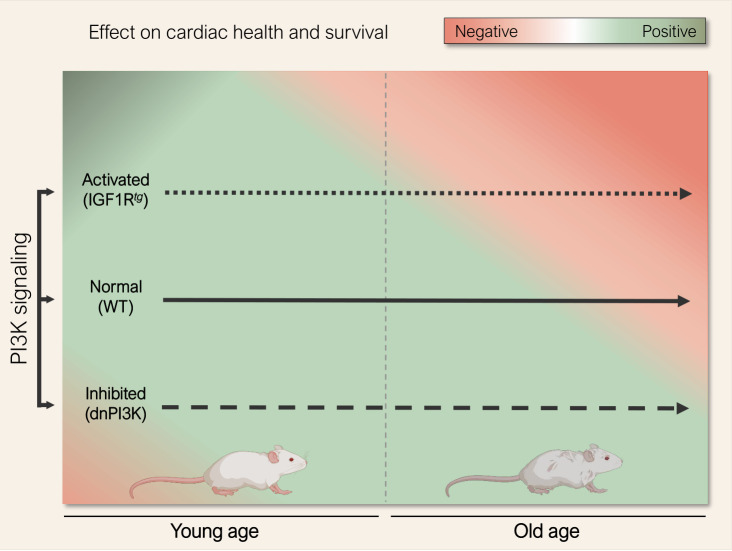
FIGURE 1: The impact of phosphoinositide 3-kinase (PI3K) signaling in regulating cardiac health and survival during the course of life. PI3K signaling is crucial for cardiac homeostasis during early life as young mice harbouring a dominant-negative mutation in the p110α isoform of PI3K (dnPI3K) exhibited subnormal cardiac performance and increased risk of mortality in early life, but extended cardiac health-span and longevity later in life (dashed arrow). Accordingly, PI3K activation in mice by cardiomyocyte-specific overexpression of human insu-lin-like growth factor 1 receptor (IGF1R^*tg*^) induced physiological left ventricular hypertrophy and conferred cardiac functional benefits at young age, but worsened cardiac health at old age, as it accelerated cardiac decline, leading to heart failure and shortened lifespan in IGF1Rtg mice (dotted arrow). Abbreviation: WT, wild type. Mouse cliparts were generated with Biorender.com.
